# Synthesis and Evaluation of [^11^C]7-Halogen-2-Phenyl Isoindolone Derivatives: Potential PET Radioligands for *in vivo* Imaging of 5-HT_2__*C*_ Receptors

**DOI:** 10.3389/fnins.2021.766320

**Published:** 2021-11-26

**Authors:** Fanxing Zeng, Jonathon A. Nye, Ronald J. Voll, Jiyoung Mun, Mark M. Goodman

**Affiliations:** ^1^Department of Radiology and Imaging Sciences, Emory University, Atlanta, GA, United States; ^2^Center for Systems Imaging, Emory University, Atlanta, GA, United States

**Keywords:** PET radioligand, 5-HT_2__*C*_ receptor, *in vivo*, brain imaging, carbon-11

## Abstract

The serotonin 5-HT_2__*C*_ receptor (5-HT_2__*C*_R) is abundantly expressed throughout the central nervous system, and involved in a variety of neuroendocrine and neurobehavioral processes. The development of a selective radioligand that will enable *in vivo* imaging and quantification of 5-HT_2__*C*_R densities represents a significant technological advancement in understanding both the normal function and pathophysiology of the 5-HT_2__*C*_R. Four 7-halogen-2-phenyl isoindolones (7-F, Cl, Br, I) were synthesized and displayed high affinities for 5-HT_2__*C*_R and high selectivity over 5-HT_2__*A*_ and 5-HT_2__*B*_. [^11^C]7-Chloro-2-[4-methoxy-3-[2-(4-methylpiperidin-1-yl)ethoxy]phenyl]isoindolin-1-one (**6**) and [^11^C]7-iodo-2-[4-methoxy-3-[2-(4-methylpiperidin-1-yl)ethoxy]phenyl]isoindolin-1-one (**9**) were synthesized in high radiochemical yield of 37–44% [*n* = 10, decay corrected from end of (^11^C)CH_3_I synthesis] with high radiochemical purity *via O*-methylation with [^11^C]CH_3_I, respectively. MicroPET imaging studies in male rats with or without 5-HT_2__*C*_ antagonist SB-242084 showed that [^11^C]**6** and [^11^C]**9** display specific bindings to 5-HT_2__*C*_R in the choroid plexus and hippocampus. *In vivo* microPET brain imaging studies in rhesus monkeys demonstrated that [^11^C]**6** and [^11^C]**9** exhibit excellent blood-brain barrier penetration. The contrast of bindings to the choroid plexus and hippocampus compared to the cerebellum peaked at 2.7 and 1.6, respectively, for [^11^C]**6**, and 3.7 and 2.7, respectively, for [^11^C]**9**, which were reduced by administration of a dose of SB-242084. Our results support the candidacy of [^11^C]**6** and [^11^C]**9** for further study as radioligands for *in vivo* quantitation of 5-HT_2__*C*_ sites by PET.

## Introduction

The serotonin (5-HT) system in the brain is directly involved in regulating various physiological actions and mental states as an important neurotransmission network which control behaviors and physical functions (Jacobs and Azmitia, 1992; Barnes and Sharp, 1999; Carhart-Harris and Nutt, 2017). Dysregulation of serotonergic function and significant alteration in serotonin receptor binding has been increasingly implicated in the pathology of physiological and psychiatric disorders (Messing and Lytle, 1977; Coccaro et al., 1989; Naughton et al., 2000; Stockmeier, 2003). The 5-HT_2__*C*_ receptor (5-HT_2__*C*_R) is one of 14 5-HT receptor subtypes that binds the endogenous neurotransmitter serotonin. Expression of the 5-HT_2__*C*_R is widely distributed in the mammalian brain with the highest concentration in the choroid plexus. Sufficient densities for imaging are also identified in the hippocampus, amygdala and hypothalamus, while low densities of 5-HT_2__*C*_R are found in the cortex and cerebellum (Hoyer et al., 1986; Clemett et al., 2000). The 5-HT_2__*C*_R has been implicated in mediating the interaction between serotonergic and dopaminergic systems. Substantial preclinical and clinical findings on 5-HT_2__*C*_R agonists and antagonists demonstrated 5-HT_2__*C*_R as a potential therapeutic target for the treatment of schizophrenia, anxiety, depression, drug abuse, and Parkinson’s disease (Millan, 2005; Heisler et al., 2007; Rosenzweig-Lipson et al., 2007; Jensen et al., 2010). Its involvement in feeding regulation and energy balance led to the FDA approval of the 5-HT_2__*C*_R agonist lorcaserin for treatment of obesity.

**FIGURE 1:**
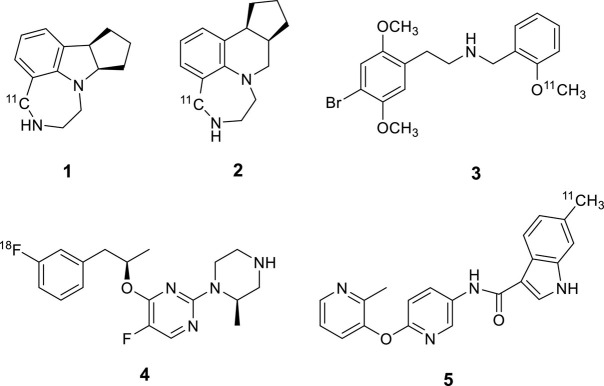
Chemical structures of reported PET imaging agents for 5-HT_2C_R.

Although PET imaging of 5-HTRs has been progressing for almost three decades, the successful radiotracers developed so far for human studies are limited to 5-HT_1__*A*_R, 5-HT_1__*B*_R, 5-HT_2__*A*_R, 5-HT_4_R and 5-HT_6_R. Several attempts for the development of 5-HT_2__*C*_R PET tracers were recently made with limited success (Figure 1). Two potent benzazepine 5-HT_2__*C*_R agonists, WAY-163909 and Vabicaserin, were labeled with carbon-11. PET imaging evaluation indicated that both [^11^C]WAY-163909 (Neelamegam et al., 2014) (**1**) and [^11^C]Vabicaserin (Neelamegam et al., 2014) (**2**) exhibited high non-specific binding in baboon brains. [^11^C]Cimbi-36 (Finnema et al., 2014) (**3**) was reported as the first agonist suitable for imaging 5-HT_2__*C*_R in the choroid plexus of the primate brain. However, [^11^C]Cimbi-36 is also a potent 5-HT_2__*A*_R agonist with Ki = 0.5 nM for 5HT_2__*A*_R vs. Ki = 1.7 nM for 5HT_2__*C*_R. This major shortcoming necessitates blocking 5HT_2__*A*_R prior to administration of [^11^C]Cimbi-36 in the quantitative assessment of 5-HT_2__*C*_R binding in CNS disorders. [^18^F]4-(3-Fluorophenethoxy)pyrimidine (**4**) was synthesized and evaluated in the rat brain (Kim et al., 2017). Although [^18^F]**4** exhibited specific binding to 5-HT_2__*C*_R, its low to moderate ratios of uptake in regions of interest to cerebellum and fast washout from choroid plexus limited its utility as a PET radiotracer for 5-HT_2__*C*_R. Recently, we evaluated a C-11 labeled pyridyloxypyridyl indole carboxamide derivative, 6-methyl-*N*-[6-[(2-methyl-3-pyridinyl)oxy]-3-pyridinyl]1*H*-indole-3-carboxamide (**5**) as a 5-HT_2__*C*_R PET imaging agent (Zeng et al., 2018). MicroPET studies in rhesus monkeys demonstrated that [^11^C]**5** displays a high level of specific binding in the choroid plexus, however, the overall brain uptake and retention of the tracer was low.

As our continuous effort to develop 5-HT_2__*C*_R-specific PET imaging agents, we selected halogen substituted isoindolones (represented by **6** and **7**) as potential target molecules based on the reported excellent *in vitro* binding profiles (Hamprecht et al., 2007). Chloro- and bromo- isoindolone derivatives (**6** and **7**) exhibited high affinity for 5-HT_2__*C*_R and high selectivity over 5-HT_2__*a*_ and 5-HT_2__*b*_ (pKi of **6** for 5-HT_2__*a*_, 5-Ht_2__*b*_, and 5-HT_2__*C*_ = 6.1, 6.9, and 8.8; pKi of **7** for 5-HT_2__*a*_, 5-Ht_2__*b*_, and 5-HT_2__*C*_ = 6.2, 6.8, and 9.1, respectively). In addition, **6** and **7** showed great brain permeability as measured by brain to blood plasma ratio. We synthesized compounds **6** and **7**, and fluoro- and iodo-isoindolone derivatives, **8** and **9**, and compared their binding affinity and selectivity at 5-HT_2__*C*_R. In addition, compounds **6** and **9** were radiolabeled with carbon-11 and PET imaging studies in rats and non-human primates were performed for evaluation as 5-HT_2__*C*_R PET radioligands.

## Results and Discussion

**SCHEME 1:**
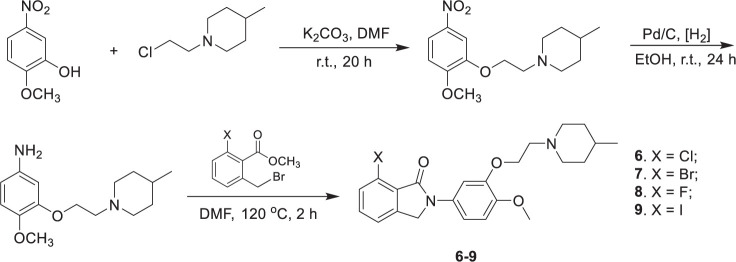
Synthesis of 7-halogen isoindolones.

**SCHEME 2:**
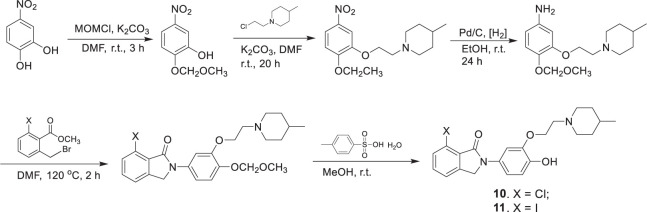
Synthesis of hydroxyphenyl isoindolone precursors for radiolabeling.

Compounds **6**–**9** were synthesized in three steps (Scheme 1), which include alkylation of the phenols with *N*-(2-chloroethyl)-4-methylpiperidine, reduction of the nitro compounds using Pd/C, and condensation of the resulting phenylamines with the corresponding 6-halogen-2-bromomethylbenzoate. The synthesis of the precursors **10** and **11** followed the same procedure, except that 2-(methoxymethoxy)-5-nitrophenol was used in the alkylation reaction. The MOM protection group was then cleaved with excess p-toluenesulfonic acid to afford the hydroxyphenylisoindolones as the radiolabeling precursors (Scheme 2).

**SCHEME 3:**
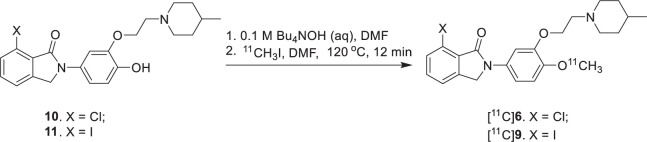
Radiosynthesis of [^11^C]**6** and [^11^C]**9**.

All compounds **6**–**9** displayed high binding affinities to 5-HT_2__*C*_ with >90% binding at a concentration of 10 μM. The 5-HT_2__*C*_ competition assay with [^3^H]mesulergine showed that iodo derivative **9** has the highest binding of 1.1 nM, while chloro and bromo derivatives **6** and **7** exhibit similar bindings of 2.2 and 2.9 nM, respectively, which is 2–3 times less than that for **9**, and fluoro derivative **8** has the lowest affinity of 28 nM. Compounds **6**, **7**, and **9** exhibit high binding selectivity over 5-HT_2__*B*_ (80–100 times) and <50% primary bindings at 5-HT_2__*A*_. In addition, no significant binding was observed in other 5-HT subtypes and D_1_-D_5_ receptors (Table 1). We selected **6** and **9** for carbon-11 radiolabelling and PET imaging studies.

**TABLE 1:** Binding affinities of 7-halogen isoindolones at serotonin (5-HT_1A_-5-HT_7_) and dopamine (D_1_-D_5_) receptors (*Ki*, nM)^a^.

**Compound**	**5-HT_2C_**	**5-HT_2A_**	**5-HT_2B_**	**5-HT_1A_**	**5-HT_5–7_**	**D_1_-D_5_**
6	2.2 ± 0.3	> 1,000	194 ± 74	> 1,000	>1,000	> 1,000
7	2.9 ± 0.7	> 1,000	177 ± 75	> 1,000	>1,000	> 1,000
8	28 ± 14	> 1,000	434 ± 204	> 1,000	>1,000	> 1,000
9	1.1 ± 0.3	> 1,000	126 ± 28	> 1,000	>1,000	> 1,000

*^a^Competition binding assays were conducted by NIMH Psychoactive Drug Screening Program (PDSP). Data are reported as means of three separate competitive experiments ± standard deviation.*

[^11^C]**6** and [^11^C]**9** were prepared *via O*-methylation of **10** and **11** with [^11^C]CH_3_I in the presence of 0.1 M Bu_4_NOH in DMF followed by HPLC purification, respectively. [^11^C]**6** and [^11^C]**9** were obtained in an average radiochemical yield of 37–44 ± 4% (*n* = 10, decay corrected) with ≥97% radiochemical and chemical purity. The total synthesis time was 50 ± 5 min end of bombardment and the specific activity were in the range of 0.5–1.2 Ci/μmol at time of injection. The lipophilicities of **6** and **9** (octanol/phosphate buffer partition) were measured. The log *P*_7.4_ values of [^11^C]**6** and [^11^C]**9** are 2.81 and 2.89, respectively, which are in the optimal range (1.0–3.0) for compounds expected to enter the brain readily.

**FIGURE 2:**
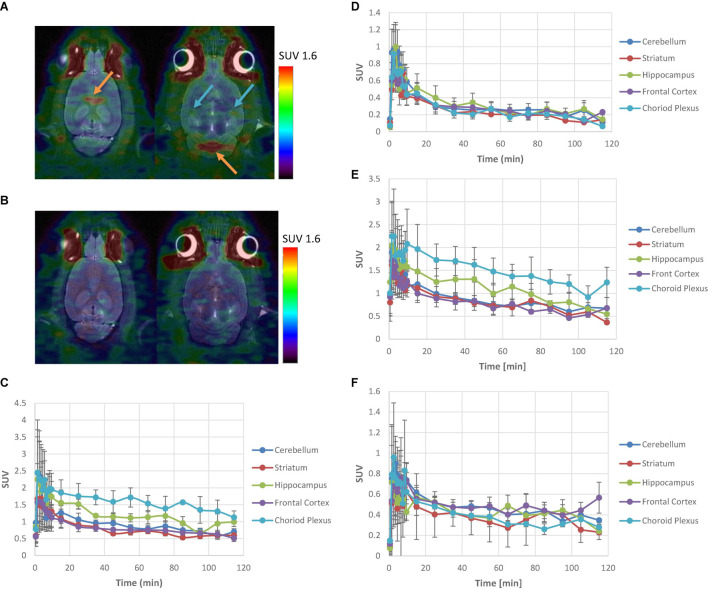
PET analysis of [^11^C]**6** and [^11^C]**9** in Sprague–Dawley rats. **(A)** Summed images of [^11^C]**6** PET/CT data from 10 to 120 min p.i. at baseline. Orange arrows depict choroid plexus; Blue arrows depict hippocampus. **(B)** Summed images of [^11^C]**6** PET/CT data from 10 to 120 min p.i. after pretreatment with 0.1 mg/kg of SB-242084. **(C)** Mean (*n* = 3) time-activity curves for brain regions of interest of [^11^C]**6** at baseline with standard error. **(D)** Mean (*n* = 3) time-activity curves for brain regions of interest of [^11^C]**6** after pretreatment with 0.1 mg/kg of SB-242084 with standard error. **(E)** Mean (*n* = 3) time-activity curves for brain regions of interest of [^11^C]**9** at baseline with standard error. **(F)** Mean (*n* = 3) time-activity curves for brain regions of interest of [^11^C]**9** after pretreatment with 0.1 mg/kg of SB-242084 with standard error.

[^11^C]**6** and [^11^C]**9** were intravenously administered to Sprague–Dawley rats and brain images were acquired with a Siemens Inveon microPET/CT imaging system, respectively (Figure 2). The regional uptake of [^11^C]**6** and [^11^C]**9** within the brain was observed in the choroid plexus and hippocampus, whereas retention in all other brain regions was low. The time-activity curves generated from imaging data showed that ratios of uptake in choroid plexus and hippocampus to that in cerebellum peaked at 1.8 and 1.2 for [^11^C]**6**, and 1.9 and 1.3 for [^11^C]**9**, respectively. Pretreating the rats with a dose of 0.1 mg/kg of SB-242084, a 5-HT_2__*C*_R antagonist (Ki at 5-HT_2__*C*_R = 3.6 nM), prior to injection resulted in a reduction of radioactivity uptake in choroid plexus and hippocampus to the same level as of the cerebellum, suggesting that the uptake of [^11^C]**6** and [^11^C]**9** in choroid plexus and hippocampus reflected specific binding.

**FIGURE 3:**
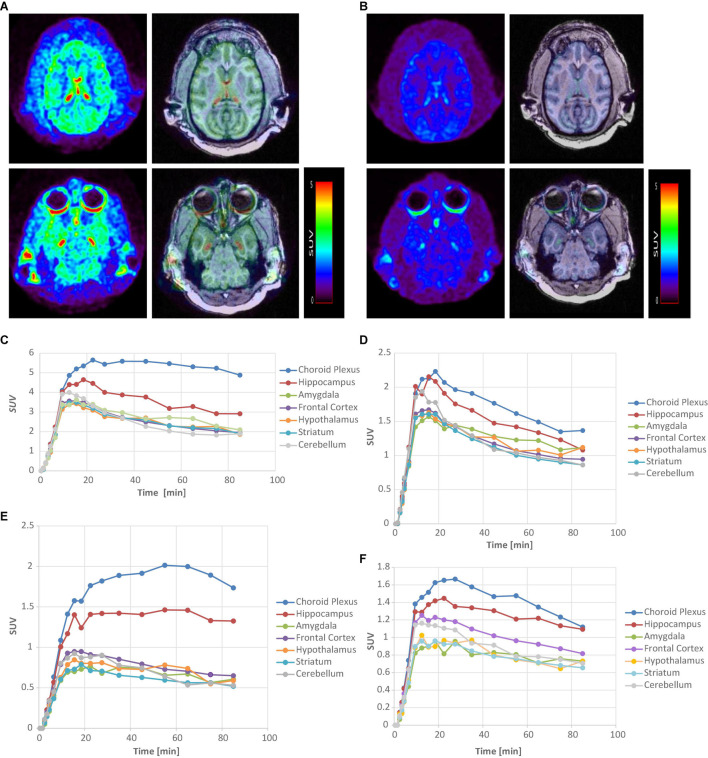
PET-MRI analysis of [^11^C]**6** and [^11^C]**9** in rhesus monkey. **(A)** MicroPET images of [^11^C]**6** (left) and corresponding fused PET/MRI images summed over all time at baseline. **(B)** MicroPET images [^11^C]**6** (left) and corresponding fused PET/MRI images summed over all time after pretreatment with 0.1 mg/kg of SB-242084. **(C)** Time-activity curves of [^11^C]**6** for brain regions of interest at baseline study. **(D)** Time-activity curves of [^11^C]**6** for brain regions after pretreatment with SB-242084. **(E)** Time-activity curves of [^11^C]**9** for brain regions of interest at baseline study. **(F)** Time-activity curves of [^11^C]**9** for brain regions after pretreatment with SB-242084.

[^11^C]**6** and [^11^C]**9** were further evaluated in rhesus monkeys using a Siemens MicroPET Focus 220 scanner (Figure 3). Contrary to the relatively low uptakes in rat brain, [^11^C]**6** and [^11^C]**9** exhibited excellent brain blood barrier penetration in the monkey. The regional uptake of [^11^C]**6** showed high uptake in the choroid plexus and hippocampus with low uptake in the cerebellum. The corresponding time-activity curves (TACs) of [^11^C]**6** showed that the radioactivity uptake in the choroid plexus peaked between 20–30 min after injection with a mean SUV value of 5.6, and the peak uptakes in the hippocampus, amygdala, frontal cortex, and cerebellum were achieved at 9.5–18.5 min postinjection. Ratios of uptake in choroid plexus, hippocampus, amygdala, frontal cortex to that in cerebellum peaked at 2.7, 1.6, 1.3, and 1.1, respectively.

Similarly, [^11^C]**9** showed high uptake in the choroid plexus and hippocampus, with low uptake in the cerebellum in the baseline study. The TACs of [^11^C]**9** indicated that the peak uptake of radioactivity in the choroid plexus and hippocampus were achieved 25–55 min after injection, and slowly washed out during the 90 min-course of PET study. The slower binding kinetics exhibited by [^11^C]**9** in comparison to [^11^C]**6** may reflect a higher binding affinity of [^11^C]**9** to the 5-HT_2__*C*_R. Although [^11^C]**9** showed lower uptakes in the choroid plexus and hippocampus than [^11^C]**6**, with SUV values of 2.01 and 1.46 vs. 5.6 and 4.6, respectively, the ratio of uptake in the choroid plexus and hippocampus compared to the cerebellum peaked higher than [^11^C]**6** at 3.7 and 2.7, respectively.

To test specific binding of [^11^C]**6** and [^11^C]**9** to the choroid plexus and hippocampus, an *in vivo* microPET blocking study was performed with 0.1 mg/kg SB-242084 30 min prior to injection, respectively. Unfortunately, due to the unexpected low solubility of SB-242084 in the injection form (10% ethanol in saline), the administered SB-242084 in soluble form to the monkeys was likely much less than the target 0.1 mg/kg. As a result, the uptake of [^11^C]**6** and [^11^C]**9** in the choroid plexus and hippocampus was still present. However, a reduction in the uptake and faster washout from the choroid plexus and hippocampus was observed compared to the baseline studies shown in Figures 3D,F. Combined with the blocking study results on rats, these results strongly suggest that PET images acquired with [^11^C]**6** and [^11^C]**9** represent the specific binding to 5-HT_2__*C*_R.

In summary, two 5-HT_2__*C*_ ligands, 7-chloro-2-[4-methoxy-3-[2-(4-methylpiperidin-1-yl)ethoxy]phenyl]isoindolin-1-one (**6**) and 7-iodo-2-[4-methoxy-3-[2-(4-methylpiperidin-1-yl)ethoxy]phenyl]isoindolin-1-one (**9**) have been synthesized, radiolabeled with carbon-11, and evaluated in rats and non-human primates. Competition binding assays demonstrated that (**6**) and (**9**) possess high 5-HT_2__*C*_ binding affinity and high selectivity over 5-HT_2__*A*_ and 5-HT_2__*B*_. *In vivo* microPET imaging studies in rats showed that [^11^C]**6** and [^11^C]**9** display specific binding to 5-HT_2__*C*_R in the choroid plexus and hippocampus. *In vivo* microPET imaging studies in monkeys demonstrated that [^11^C]**6** and [^11^C]**9** exhibited high brain uptake with specific binding to the choroid plexus and hippocampus. The high uptakes in the choroid plexus and hippocampus to cerebellar ratios in non-human primates strongly support the candidacy of [^11^C]**6** and [^11^C]**9** for further study as radioligands for *in vivo* quantitation of 5-HT_2__*C*_ sites by PET.

## Data Availability Statement

The original contributions presented in the study are included in the article, further inquiries can be directed to the corresponding author/s.

## Ethics Statement

The animal study was reviewed and approved by Institutional Animal Care and Use Committee (IACUC), Emory University.

## Author Contributions

FZ and MG contributed to the design of the study. FZ performed the synthesis and radiolabelling. RV and JM contributed to the radiolabeling. JN performed the imaging process. All authors contributed to manuscript revision, read, and approved the submitted version.

## Conflict of Interest

The authors declare that the research was conducted in the absence of any commercial or financial relationships that could be construed as a potential conflict of interest.

## Publisher’s Note

All claims expressed in this article are solely those of the authors and do not necessarily represent those of their affiliated organizations, or those of the publisher, the editors and the reviewers. Any product that may be evaluated in this article, or claim that may be made by its manufacturer, is not guaranteed or endorsed by the publisher.

## References

[B1] Barnes N. M. Sharp T. A. (1999). A review of central 5-HT receptors and their function. *Neuropharmacology* 38 1083–1152. 10.1016/s0028-3908(99)00010-6 10462127

[B2] Carhart-Harris R. L. Nutt D. J. (2017). Serotonin and brain function: a tale of two receptors. *J. Psychopharmacol.* 31 1091–1120. 10.1177/0269881117725915 28858536 PMC5606297

[B3] Clemett D. A. Punhani T. Duxon M. S. Blackburn T. P. Fone K. C. F. (2000). Immunohistochemical localization of the 5-HT_2C_ receptor protein in the rat CNS. *Neuropharmacology* 39 123–132. 10.1016/s0028-3908(99)00086-6 10665825

[B4] Coccaro E. F. Siever L. J. Klar H. M. Maurer G. Cochrane K. Cooper T. B. (1989). Serotoninergic studies in patients with affective and personality disorders. *Arch. Gen. Psychiatry* 46 587–599. 2735812 10.1001/archpsyc.1989.01810070013002

[B5] Finnema S. J. Stepanov V. Etrup A. Nakao R. Amini N. Svedberg M. (2014). Characterization of [^11^C]Cimbi-36 as an agonist PET radioligand for the 5-HT_2A_ and 5-HT_2C_ receptors in the non-human primate brain. *Neuroimage* 84 342–353. 10.1016/j.neuroimage.2013.08.035 23994452

[B6] Hamprecht D. Micheli F. Tedesco G. Checchia A. Donati D. Petrone M. (2007). Isoindolone derivatives, a new class of 5-HT_2C_ antagonists: synthesis and biological evaluation. *Bioorg. Med. Chem. Lett.* 17 428–433. 10.1016/j.bmcl.2006.10.029 17074479

[B7] Heisler L. K. Zhou L. Bajwa P. Hsu J. Tecott L. H. (2007). Serotonin 5-HT(2C) receptors regulate anxiety-like behavior. *Genes Brain Behav.* 6 491–496. 10.1111/j.1601-183x.2007.00316.x 17451451

[B8] Hoyer D. Pazos A. Probst A. Palacios J. M. (1986). Serotonin receptors in the human brain. II. Characterization and autoradiographic localization of 5-HT1C and 5-HT2 recognition sites. *Brain Res.* 376 97–107. 10.1016/0006-8993(86)90903-0 2941113

[B9] Jacobs B. L. Azmitia E. C. (1992). Structure and Function of the Brain Serotonin System. *Physiol. Rev.* 72 165–229. 10.1152/physrev.1992.72.1.165 1731370

[B10] Jensen N. H. Cremers T. I. Sotty F. (2010). Therapeutic potential of 5-HT_2C_ receptor ligands. *Sci. World J.* 10 1870–1885. 10.1100/tsw.2010.180 PMC5763985 20852829

[B11] Kim J. Moon B. S. Lee B. C. Lee H. Kim H. Choo H. (2017). A potential PET radiotracer for the 5-HT2C receptors: synthesis and in Vivo evaluation of 4-(3-[^18^F]fluorophenethoxy)pyrimidine. *ACS Chem. Neuosci.* 8 996–1003. 10.1021/acschemneuro.6b00445 28194935

[B12] Messing R. B. Lytle L. D. (1977). Serotonin-containing neurons: their possible role in pain and analgesia. *Pain* 21 1–21. 10.1016/0304-3959(77)90083-5 22060

[B13] Millan M. J. (2005). Serotonin 5-HT2C receptors as a target for the treatment of depressive and anxious states: focus on novel therapeutic strategies. *Therapie* 60 441–460. 10.2515/therapie:2005065 16433010

[B14] Naughton M. Mulrooney J. B. Leonard B. E. (2000). A review of the role of serotonin receptors in psychiatric disorders. *Hum. Psychopharmacol.* 15 397–415. 10.1002/1099-1077(200008)15:6<397::aid-hup212>3.0.co;2-l 12404302

[B15] Neelamegam R. Hellenbrand T. Schroeder F. A. Wang C. Hooker J. M. (2014). Imaging evaluation of 5HT_2C_ agonists, [^11^C]WAY-163909 and [^11^C]Vabicaserin, formed by Pictet-Spengler cyclization. *J. Med. Chem.* 57 1488–1494. 24491146 10.1021/jm401802f PMC3983360

[B16] Rosenzweig-Lipson S. Dunlop J. Marquis K. L. (2007). 5-HT2C Receptor agonists as an innovative approach for psychiatric disorders. *Drug News Perspect.* 20 565–571. 10.1358/dnp.2007.20.9.1162244 18176661

[B17] Stockmeier C. A. (2003). Involvement of serotonin in depression: evidence from postmortem and imaging studies of serotonin receptors and the serotonin transporter. *J. Psychiatr. Res.* 37 357–373. 12849929 10.1016/s0022-3956(03)00050-5

[B18] Zeng F. Nye J. A. Voll R. J. Howell L. Goodman M. M. (2018). Synthesis and Evaluation of Pyridyloxypyridyl Indole Carboxamides as Potential PET Imaging Agents for 5-HT_2C_ Receptors. *ACS Med. Chem. Lett.* 9 188–192. 10.1021/acsmedchemlett.7b00443 29541358 PMC5846131

